# *Amesia nigricolor*, a novel endophyte of *Encephalartos bubalinus*, exhibiting a robust taxol biosynthetic stability: chemical characterization and biological activities

**DOI:** 10.1186/s12934-025-02827-5

**Published:** 2025-09-03

**Authors:** Asmaa Gamal, Ashraf S. A. El-Sayed, Eman Fikry, Nora Tawfeek, Azza M. El-Shafae, Maher M. El-Domiaty

**Affiliations:** 1 Pharmacognosy Department, Faculty of Pharmacy, Zagazig University, Zagazig, 44519 Egypt; 2 Enzymology and Fungal Biotechnology Lab (EFBL), Botany and Microbiology Department, Faculty of Science, Zagazig University, Zagazig, 44519 Egypt

**Keywords:** *Amesia nigricolor*, *Encephalartos bubalinus*, Taxol, Anticancer, Anti-wound healing activity

## Abstract

Diminishing the productivity of Taxol by the potential fungi with storage is the key hurdle that impedes their applications to be an industrial platform for Taxol production. Thus, exploring of a fungal isolate with a reliable robustness for Taxol biosynthesis is the objective of this study. Although, *Encephalartos bubalinus* has diverse ethnopharmaceutical properties, however, the identity of its endophytic fungi remains poorly explored. Therefore, the endophytic fungi inhabiting this plant has been isolated and characterized, and their Taxol productivity was assessed. *Amesia nigricolor* OR364127.1*,* an endophyte of *E. bubalinus,* was characterized as the potent biologically active and Taxol producer (105 μg/l). The sample identity was resolved from the HPLC, FT-IR and MS/MS analysis, with the molecular mass/ fragmentation pattern was identical to authentic one. The extracted Taxol of *A. nigricolor* had a strong activity against the HepG2 (IC_50_ 19 nM) and MCF7 (IC_50_ 23 nM) with a selectivity index 13.2 and 11.9 to the normal Vero cells. Taxol of *A. nigricolor* had a powerful anti-wound healing, and apoptotic properties, with ability to stop the G2/M cell cycle, ensuring their consistent biological activity to the authentic one. The Taxol yield by *A. nigricolor* was enhanced by 2 folds (205.2 μg/l), with the statistical bioprocessing by CCD. The half-life time for production of Taxol by *A. nigricolor* was more than 10 months, that being higher than those reported for various Taxol-producing fungi, ensuring the relative stability of the biosynthetic machinery of Taxol by *A. nigricolor* with storage as solid cultures at 4°C. A relative restoring to the Taxol productivity by *A. nigricolor* was noticed with ethylacetate extract of *E. bubalinus,* ensuring the presence of chemical signals inducing Taxol productivity by *A. nigricolor*. To the best of our knowledge, this is the first recorded endophytic fungus “*A. nigricolor* EFBL-AG” with a relative stability of Taxol biosynthetic machinery.

## Introduction

Cancer diseases are one of the leading causes of human mortality, leading to higher clinical, social, and economic burdens with a direct relation to a specific Disability-Adjusted Life Years [1]. Among the different chemotherapeutics drugs, Taxol “diterpenoids” is one of the blockbuster anticancer compound against several tumors types such as breast, lung, colon, liver carcinoma as approved by FDA in 1994 [2, 3]. The authoritative activity of Taxol was due to its unique affinity for β-tubulin binding, thus stimulating the microtubule assembly, consequently halting the cellular mitotic division by stopping their division at G2/M phase [4, 5]. Taxol was explored from the bark of *Taxus brevifolia* in 1970, was chemically and biologically characterized as a powerful lead anticancer compound [6]. However, the lower Taxol yield, vulnerability of *T. brevifolia* to the environmental fluctuations, and reproducibility, making the global dependence on this source is a challenging [7, 8]. Consequently, the exploring of novel sources of Taxol with higher yield has become the alternative route to fulfil the clinical requirements for this lead compound. Fungal endophytes possessing the Taxol-producing potency was emerged as an affordable approach for industrial production of Taxol for their feasibility of growth, resilience to climatic variations, and amenability to molecular manipulation [9, 10]. Plethora of the fungal endophytes inhabiting the different plants was reported to have the metabolic potency to produce Taxol [11–15]. However, reducing the Taxol biosynthetic potency by the fungal storage is the main challenge that halts the more employment of the fungal-based approach industrially [16–19]. The biosynthesis of fungal Taxol is encoded by a cluster of gene on the different chromosomes of the fungus, and the expression of this cluster becomes silence at normal laboratory conditions that could be ascribed to the weakening of the signals that harmonize the expression of the Taxol-encoding genes [19–23]. So, searching for a stable Taxol-producing fungus from the ethnopharmacological plants become a crucial objective. This is particularly relevant since medicinal plants represent a valuable reservoir for such novel endophytic fungi [24, 25]. The genus *Encephalartos* has been recognized with its ethnopharmaceutical properties and its diverse biologically active compounds [26], however, the endophytic fungi inhibiting this plant remain poorly understood. *Encephalartos* is the second-largest genus in Zamiaceae, comprising about 66 species found south of the Sahara Desert, with numerous species distributed throughout tropical Africa and South Africa [27]. *Encephalartos* species have recently demonstrated with their potential antimicrobial, antioxidant, and cytotoxic properties [28–30]. *Encephalartos whitelockii* served as a source of the fungal endophyte *Aspergillus niger* with reliable Taxol-producing potency; however, the attenuation of its Taxol biosynthetic potency during storage remains challenging [13]. So, the endophytic fungi inhabiting the genus *Encephalartos* “with various ethnopharmaceutical activities” could be a novel platform for a plausible and sustainability Taxol producer.

*Encephalartos bubalinus* is a rare cycad that occurs in Kenya and Tanzania, with oblanceolate pinnate leaves, arranged in a crown at the apex of the stem [31]. Despite the ethnopharmaceutical properties of *E. bubalinus,* the endophytic fungal blueprint inhabiting this plant was poorly studied, as revealed by the literature. Therefore, this work was an endeavor to explore novel fungi from *E. bubalinus* with a reliable Taxol productivity and biosynthetic stability.

## Material and methods

### Plant collection, isolation, and identification of fungi

The leaves samples of *E. bubalinus* were collected in March/2022 from El-Abd Garden in Giza, Egypt. The plant was kindly identified by Dr. Therese Labib, a Plant Taxonomist at Orman Botanical Garden in Giza, Egypt, and deposited as a voucher sample with ID# ZU-Ph-Cog-609 at Zagazig University. The leaves were surface-sterilized, rinsed with sterile distilled water used as the source for the entophytic fungi [12] on potato dextrose agar (PDA) [12, 32]. The effectiveness of surface sterilization of the plant parts was assessed by inoculating 500 μl of the washing water to the potato dextrose agar plate, then incubation, and observing the developing fungal colonies. After 10 days of incubation of the cultures at 30°C, the emerged fungi were identified based on their microscopical traits [33–36].

The potent fungi were recognized based on their ITS sequence [37, 38]. The DNA was extracted by CTAB solution, for the PCR reaction, with primers ITS5 5ʹ-TCCTCCGC-TTATTGATATGC-3ʹ and ITS4 5ʹ-GAAGTAAAAGTCGTAACAAGG-3ʹ [39]. The PCR mixture with TOPsimple™ (Cat.# P510T), gDNA, primers (5 pmol), and the thermal cycler was programmed according to our previous study [13]. The amplification products were checked by 1.5% agarose, sequenced and the retrieved sequence was BLAST searched, aligned by ClustalW algorithm with MEGAX package, and the phylogenetic relatedness was conducted with the neighbour-joining of 500 bootstrap replicates [40].

### Screening for Taxol production

The recovered fungi of *E. bubalinus* were grown on potato dextrose broth (PDB) [12, 14], for 10 days at 30°C, filtered, and extracted with ethyl acetate [13, 41]. Three biological replicates were prepared in parallel. The organic phase was dried, dissolved in methanol [14], and checked by the pre-coated TLC plates (Silica gel 60 F254), using methylene chloride/methanol/dimethyl formamide (90:9:1 v/v/v) [13, 42]. The plate was visualized by at λ_254_ nm, the spots with the same color and mobility of the authentic one (Cat. # T7402), were selected, and their intensities were measured by ImageJ software package. The recognized silica gel spots were scratched, Taxol was eluted, and determined by HPLC using RP-C18 column (Cat.#959963–902), methanol: acetonitrile: water as mobile phase (25:35: 40, v/v/v) for 20 min at 1.0 ml/min [12, 13]. The eluted fractions were detected at λ_227_ nm, Taxol yield was determined, normalized to the authentic [13, 43]. The rate-limiting gene taxa-4(5),11(12)-diene synthase (tds) of Taxol biosynthesis was assessed by PCR mining as described by our previous studies [11, 44, 45].

### Spectroscopic analyses of Taxol

The recognized Taxol samples of the selected fungi were measured by UV–Vis spectrophotometry (Shimadzu UV-1800 series) over a wavelength 200–400 nm, related to the authentic one, with methanol as a blank [13, 14]. The FT-IR spectral analysis was conducted (JASCO-FTIR 6100 Spectrophotometer) with KBr pellets at 4000–500 cm^−1^. The chemical structure of Taxol sample was explored from the LC–MS/MS (aQ C18 column), with a positive ion mode [12, 13]. The molecular mass and fragmentation of sample was determined, related to the authentic Taxol.

### Antiproliferative and antifungal activities

The cytotoxic activity of the sample against the hepatocellular HepG2 (ATCC HB-8065), breast MCF7 (ATCC HTB-22), and intestinal Caco-2 (ATCC HTB-37) tumor cell lines, in addition to the Vero cells (ATCC CCL-81), was estimated by the MTT [46]. The plate was inoculated with 10^6^ cells/ well, incubated overnight at 37°C, the tested compound was added at the desired concentrations, and re-incubated for 2 days at 37°C in 4% CO_2,_ then the MTT solution was included, incubated for 6 h, absorbance was assessed at λ_570_ nm, and the IC_50_ values were determined. The CC_50_ value for the Vero normal cells was calculated by the drug dose, halting the growth of cells by 50%, normalized to zero drug. The activity of resolved target compound was determined against *Aspergillus flavus* [13, 47, 48], as a human pathogen. The spores were seeded to the PDA, incubated for 6 h, amended with different concentrations (0.1− 10 µg/mL) of the TLC-purified sample, then re-incubated at 28°C for 5 days using 1% DMSO as control. Three biological triplicates were considered, the results were represented by mean ± SD [13].

### Anti-wound healing activity

The activity of the resolved Taxol against the wound healing of MCF-7, HepG-2 cell lines was evaluated. The microtiter plate with media was inoculated with 4 × 10^6^ cells, grown to be a confluent monolayer, a central scratch was made, the plate was washed with phosphate buffered saline, inoculated with Taxol at IC_25_ value in a fresh medium, using 2% DMSO (control). The cultures were incubated at 37 °C in 4% CO_2_ incubator, and the closure of the wound was observed and imaged. The efficiency of healing was measured as the percentage of scratch area of cells due to the tested compound, normalized to the control cells.

### Apoptosis and cell cycle analyses

The apoptotic analysis of HepG-2 cells was calculated by flow cytometry analysis with Annexin-apoptosis Kit (Cat. # K101-25). The cells were inoculated at 10^8^ cells/ well of the 96-well plate, amended with sample, incubated for one day, then the cells were gathered, washed by PBS, then amended with Annexin V-FITC-PI. After 15 min of incubation in dark, the Annexin-PS complex was flow cytometrically detected (Ex, λ_488_ nm; Em, λ_530_ nm) [49, 50]. The DNA contents of HepG-2 treated with Taxol was determined by PI (Cat #.ab139418). The plate was amended with the cells, incubated for 12 h at 37 °C, and the compound was added at its IC_25_, and incubated for two days at the same conditions. The cells were harvested, fixed in ethanol, hydrated with PBS, stained by PI solution for 30 min, and the G0/G1, S and G2/M cell percentage were evaluated from DNA contents by flow cytometry at Ex λ_493_ nm, and Em λ_636_ nm.

### Bioprocessing of Amesia nigricolor for optimizing their Taxol productivity

The nutritional requirements for optimizing the Taxol yield by *A. nigricolor* were determined by Plackett–Burman statistical design [12, 13, 38], and the parameters were symbolized by + 1 and − 1 levels (Table 2). The nutritional optimization for maximizing the enzyme yield by the response surface methodology was reported as a reliable approach to evaluate the consequences of independent parameters interactions on Taxol production, according to the first-ordered polynomial model as follow:

$$ {\text{Y }} = \, \beta 0 \, + \, \Sigma \, \beta {\text{i Xi}} $$

Y, the predicted amount of Taxol; Xi, independent variable; βi, linear coefficient and β0, the intercept of model. Biological triplicates were used for trial. The prominent variables controlling Taxol productivity by *A. nigricolor*, were further optimized by Central Composite Design [13, 51]. The 2nd ordered polynomial equation summarizing the conditions for Taxol production as follow:

$$ {\text{Y }} = \, \beta 0 \, + \, \Sigma \beta {\text{iXi }} + \hbox{-}\Sigma \beta {\text{iixii }} + \hbox{-}\Sigma \beta {\text{ijXij}}, $$

Βi, variables regression coefficient; βii, square effects regression coefficient, βij interactions regression coefficient.

### Productivity of Taxol by A. nigricolor with storage

The productivity of Taxol by *A. nigricolor* was assessed with the fungal storage by preserving the original culture at 4°C for 10 months, and the Taxol yield was assessed peridically as mentioned above. After cultural incubation, Taxol was purified, quantified by HPLC [9, 13]. The influence of organic extracts of *E. bubalinus*; ethylacetate, ethanol, dichloromethane, on restoring the Taxol productivity by *A. nigricolor,* was assased. Five grams of the *E. bubalinus* leaves were pulverized in 50 ml solvent, stored at 4°C for 12 h, filtered, dried to 10 ml, added to the 5-day-old cultures of *A. nigricolor* at different concentrations. After 14 days of incubation of culture, Taxol was assssed as described above. Blank (plant extracts without *A. nigricolor*) and control (*A. nigricolor* cultures without extract) were used.

### Fungal deposition

The sequence of ITS domian of *Amesia nigricolor*, endophyte of *E. bubalinus*, was deposited into the Genbank of accession # OR364127.1.

### Statistical analysis

Three biological replicates of each experiment were conducted, and the data were expressed by means ± SD. The statistical analyses were performed by CoStat software version 6.4. The least significant differences (LSD) were represented by the means at p ≤ 0.05 by ANOVA (analysis of variance).

## Results

### Isolation, preliminary biological activity, and screening for Taxol prodcing fungal endophytes of Encephalartos bubalinus

Seven endophytic fungal isolates were collected from *E. bubalinus* leaves on PDA media. These isolates were morphologically categorized into five genera according to their microscopic features; *Aspergillus ochraceus, A. niger, A. oryzae, Amesia nigricolor, Cladosporium* sp*, Fusarium oxysporum,* and *Alternaria alternata* as summraized in Table 1. The fungal islates were grown on PDB medium, under standrad conditions, the cultural metabolites were extracted, and their biological activity was calculated by well-diffusion assay using *A. flavus*. From the results, the crude extrcats of the tested fungi exhibited a variable activities towards *A. flavus,* in concentration-dependent paradigm. Extract of ethylacetate of *A. nigricolor* showed the superior inhibitory effect towards *A. flavus*, with a 25 mm zone of inhibition, followed by *A. oryzae* (22 mm diamter of inhibition zone), *F. oxysporum* and *A. ochraceus* (21–19 mm). The antifungal activity of the tested compounds was used as a preliminary sign to their antiproliferative activity. Taxol productivity of the isolated endophytic fungi of *E. bubalinus* leaves was evaluated in PDB, after culture incubation at standrad conditions, Taxol was extracted and qunatified. The putative sample spots on TLC that exhibited a similar mobility and color of the standard one at λ_254_ nm UV illumination were considered. *Amesia nigricolor* had the highest Taxol yield (105.2 μg/L), *Aspergillus oryzae* (88.5 μg/L), *A. alternata* (45.6 μg/L) and *A. ochraceus* (25.9 μg/L), in contrary to the undetectable Taxol producing potency of *A. niger*, *Cladosporium* sp, and *Fusarium oxysporum* (Table 1), as evaluated from the HPLC analyses. The amount and purity of the puified Taxol were confirmed from the HPLC, giving a distinict peak at 4.4 min, that was completely similar to the authentic one.

**Table 1:** Screening for Taxol production by the recovered endophytic fungal isolate of *Encephalartos bubalinus*

No	Fungal isolate	Taxol Conc. (μg/L)	
		HPLC	TLC
1	*Aspergillus ochraceus*	25.9 ± 1.89	21.0 ± 1.9
2	*Amesia nigricolor*	105.2 ± 2.55	88.5 ± 2.1
3	*Cladosporium* sp	0	0
4	*Aspergillus oryzae*	88.5 ± 3.25	75.9 ± 2.7
5	*Fusarium oxysporum*	0	0
6	*Alternaria alternata*	45.6 ± 5.22	40.3 ± 3.4
7	*Aspergilllus niger*	0	0

### Morphological and molecular identification of the selected Taxol-producing fungi

**Fig. 1:**
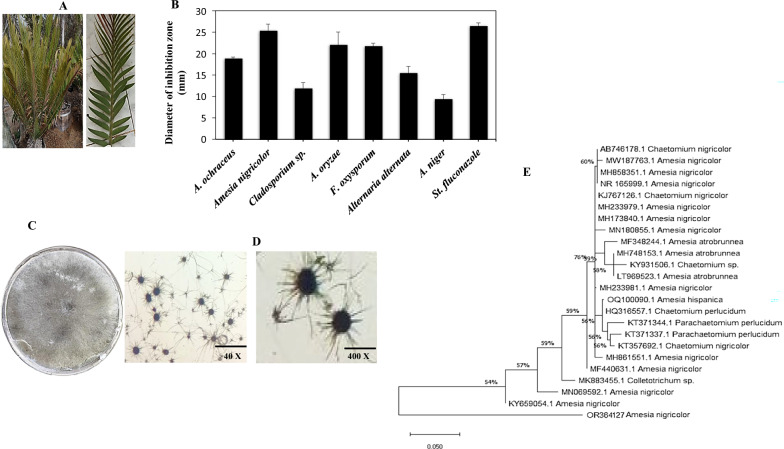
**A** Morphological view of *E. bubalinus* leaves.** B** Antifungal activity of the crude ethylacetate of the endophytic fungal isolates of *E. bubalinus* against *A. flavus* as model of human fungal pathogen, at 10 μg/ml.** C** Plate cultures of the most potent Taxol producing fungal isolate.** D** Ascocarp “Ascomata” of the potent isolate at 40 X and 400 X by light microscope.** E** Molecular phylogenetic analysis of fungus based on its ITS sequence, regarding to the database deposited sequences by the Maximum Likelihood method

**Fig. 2:**
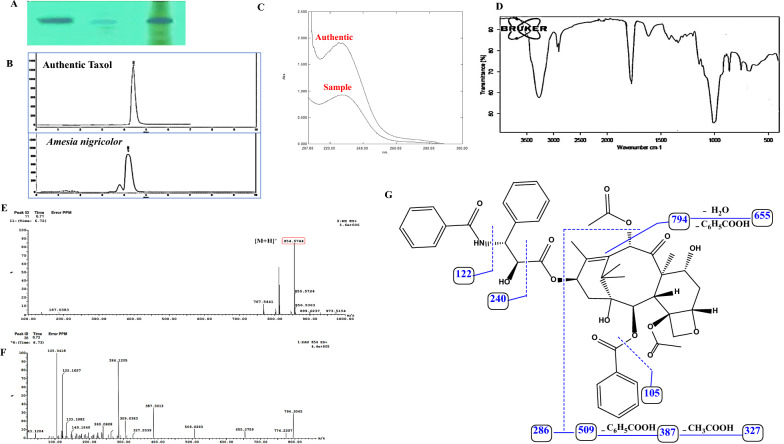
Chemical analysis of extracted Taxol from *A. nigricolor*. **A** The TLC plate of the extracted Taxol normalizing to authentic Taxol. **B** HPLC chromatogram of the TLC purified Taxol from *A. nigricolor*, compared to the authentic one at retention time 4.4 min. UV- spectrum **C** and FT-IR spectrum **D** of the extracted *A. nigricolor* Taxol. LC–MS chromatogram **E**, and LC–MS/MS fragmentation **F** of the extracted Taxol. **G**, Scheme of fragmentation pattern of Taxol by the 2nd MS

The selected isolates were recognized relied on its morphological traits. The fungal spores were centrally inoculated to the PDA medium, incubated for 10 days at 30 °C, the mycelium was microscopically inspected. The fungus has a superficial ascomata, ostiolate, olivaceous grey, subglobose, brown color ascomatal wall, loosely coiled terminal hairs, apically circinate, appearaning a loosely packed young ascomata with hairlike appendages [33], as shown in Fig. 1. These micrscopical features were typically consistent with those of the *Amesia nigricolor* (Ames) (Basionym: *Chaetomium nigricolor* Ames, Synonym: *Chaetomium amberpetense*). The isolate, *A. nigricolor*, was authenticated relied on their ITS sequence of rRNA, using gDNA as a PCR template, giving a 520 bp amplicon, as shown in Fig. 2. This amplicon was sequenced, annotated and deposited to the genbank OR364127.1. The ITS sequences of *A. nigricolor* EFBL-AG OR364127.1 showed 99.6% similarity with the isolates of *A. nigricolor* MN180855, MN069592, KT357692, KJ767126, MW187763, MH861551, MH173840 and MH233979 with 98% query coverage and zero E value.

### Spectroscopic analysis of purified Taxol from A. nigricolor

The Taxol samples extracted from the TLC plates, was authenticated from HPLC, FT-IR, and LC–MS/MS appraoches. The purified sample of Taxol of *A. nigricolor* had identical retention time of the authentic one (5 min), as revealed from the HPLC chromatogram (Fig. 2). As well as, the purified *A. nigricolor* Taxol sample had a distinctive maximum absorption at λ_227_ nm, identical to the authentic (Fig. 2).

From the FTIR analysis, the sample had a peak at 3430 to 3350 cm^–1^ for stretching of the hydroxyl, amide groups, in addition to stretching of aliphatic CH groups in range of 2935 to 2855cm^–1^. The peaks at 1730 and 1604 cm^−1^ were nominated to the ester groups stretch and aromatic ring stretch. The carboxyl group stretch frequency had a maximum absorbance at 1266 cm^–1^, the stretching at 1022 cm^–1^ was attributed to the alkyl C-O of ester bond. The highest peaks at 1020.01 cm^–1^ was atributed to the the aromatic C and H bends were (Fig. 2). From FT-IR analysis, the Taxol sample has the same functional groups and stretches of the standard Taxol, authenticating the structure of the target compound as Taxol.

The LC/MS spectra explpored that Taxol exhibited a molecular mass m/z 854.5, as depicted in Fig. 2, that consistent with the molecular fragmentation pattern observed in standard Taxol as well as Taxol of *T. brevifoliia* and *T. baccata* [52]. The molecular ion peak of Taxol was detected at m/z 854.5 and MS/MS base peak ion at m/z 286. The other characteristic MS^2^ fragment ions 749, 655, 509, 387, 327, 122, and 105 ensure the target compound's chemical identity as Taxol [13]. Overall, the previous results have confirmed the structure of the purified compound as Taxol.

### Biological activities of the purified Taxol of A. nigricolor

**Fig. 3:**
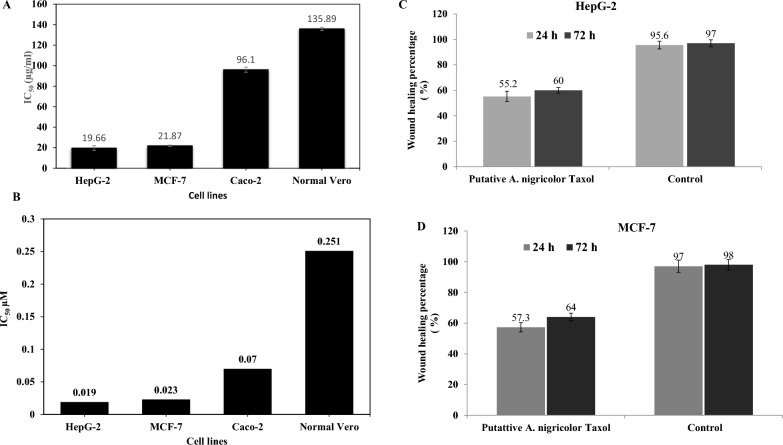
The cytotoxic and wound healing activities antiproliferative activities of the extracted Taxol and crude ethyl acetate extracts of *A. nigricolor.* The activity of the crude ethyl acetate extracts (**A**), and the putative extracted Taxol (**B**) of *A. nigricolor* towards the tumor cell lines HepG-2, MCF-7, and Caco-2, compared to the normal Vero cells, as revealed from the IC_50_ values. Taxol samples were purified from the TLC silica gel spots, their purity was checked by the HPLC, and their anti -wound healing activity was assessed towards the HepG-2 (**C**) and MCF-7 (**D**) cell lines

The biological activity of the purified sample of *A. nigricolor* was determined against different tumor cell lines, related to Vero cells. The cytotoxic, wound healing activities of the extracted Taxol and crude ethyl acetate extracts of *A. nigricolor* was shown in Fig. 3. From the cells viability (Fig. 3), the ethylacetate extract of *A. nigricolor* had a strong effect against HepG-2 (IC_50_ 19.6 μg/ml), MCF-7 (IC_50_ 21.8 µg/ml) and Caco-2 (IC_50_ 96.1 μg/ml), regarding to Vero cells. As well as, selectivity index of *A. nigricolor* ethylacetate extracts towards HepG2 and MCF7 cells was 6.9 and 6.2, compared to the normal cells. Taxol of *A. nigricolor* demonstrated a substantial efficiency against the experimented cell lines. Taxol has a strong activity against HepG2 (IC_50_ 0.019 μM), MCF7 (IC_50_ 0.021 μM) and Caco-2 cells (IC_50_ 0.07 μM). The CC_50_ concentration of the extracted Taxol for the Vero cells was 0.251 μM. The selectivity indices of the purified Taxol were 13.2, 11.9 and 3.6 towards HepG2, MCF7 and Caco2 cells, respectively, revealing the Taxol selectivity in combating cancer cells, than normal cells.

The impact of extracted *A. nigricolor* Taxol on migration of HepG-2 and MCF7 cells was considered by quantifying the gap closure after 72 h, normalized to the control ones. The scratch closure was significantly inhibited by *A. nigricolor* Taxol over time regarding to the control cells, with the time. Compared to the zero drug cells, the wound healing % of the homogeneous monolayers of HepG2 and MCF-7 was around 55–57% after 24 h, and 60–64% after 72 h (Fig. 3).

### Apoptosis and cell cycle analysis

**Fig. 4:**
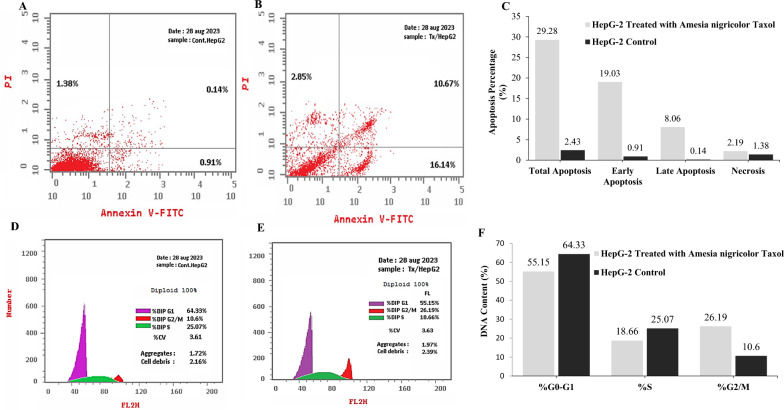
Apoptotic and cell cycle analyses of the HepG-2 cells by Flow cytometry with Annexin V-FITC. The cells were exposed to IC_25_ value of Taxol, and the apoptosis was measured after 48 h of incubation. Apoptotic analysis of HepG-2 -cells without treatment (**A**), and in response to treatment with Taxol (**B**), and the overall quantitative results of apoptosis (**C**). The cell cycle analysis of the HepG-2 without treatment (**D**), and in response to treatment with Taxol (**E**) and overall quantitative analysis of cell cycle (**F**)

Taxol extracted from *A. nigricolor* showed a strong activity against HepG-2 cells. So, the apoptosis and cell cycle of these cells for Taxol has been determined by Annexin V-PI. The purified Taxol of *A. nigricolor* demonstrated a noticeable effect on inducing the apoptotic process of HepG-2 cells, into early and late apoptotic stages normalized to the control. The early, late apoptosis, and necrosis of HepG-2 cells were increased by about 19.03, 8.06, and 2.19%, respectively, with the Taxol of *A. nigricolor*. However, the control cells exhibited an early, late apoptosis and necrosis by 0.91, 0.14, and 1.38%, as shown in Fig. 4. So, Taxol treatment increased the ratio of total apoptosis of HepG-2 by 12 folds related to the control. The cell cycle of HepG-2 for treatment with Taxol of *A. nigricolor* has been assessed, by amending Taxol at IC_25_ value, incubating the cells at standard conditions, and measuring the DNA contents by PI, G0/G1, S and G2/M phases of the cells were estimated. The maximum arrest of cellular growth of HepG-2 due to the treatment of Taxol, was observed at G2/M phase, normalized to Vero cells, as revealed from the DNA contents that were 26.1 and 10.6% for the HepG-2 and Vero cells, respectively (Fig. 4). The purified Taxol *A. nigricolor* has no a remarkable impact on cell cycle of HepG2 at G0/G1 and S-phases, with a substantial halting effect on cell cycle at the G2/M phase.

### Bioprocessing of A. nigricolor for maximizing the Taxol productivity by response surface methodology

**Fig. 5:**
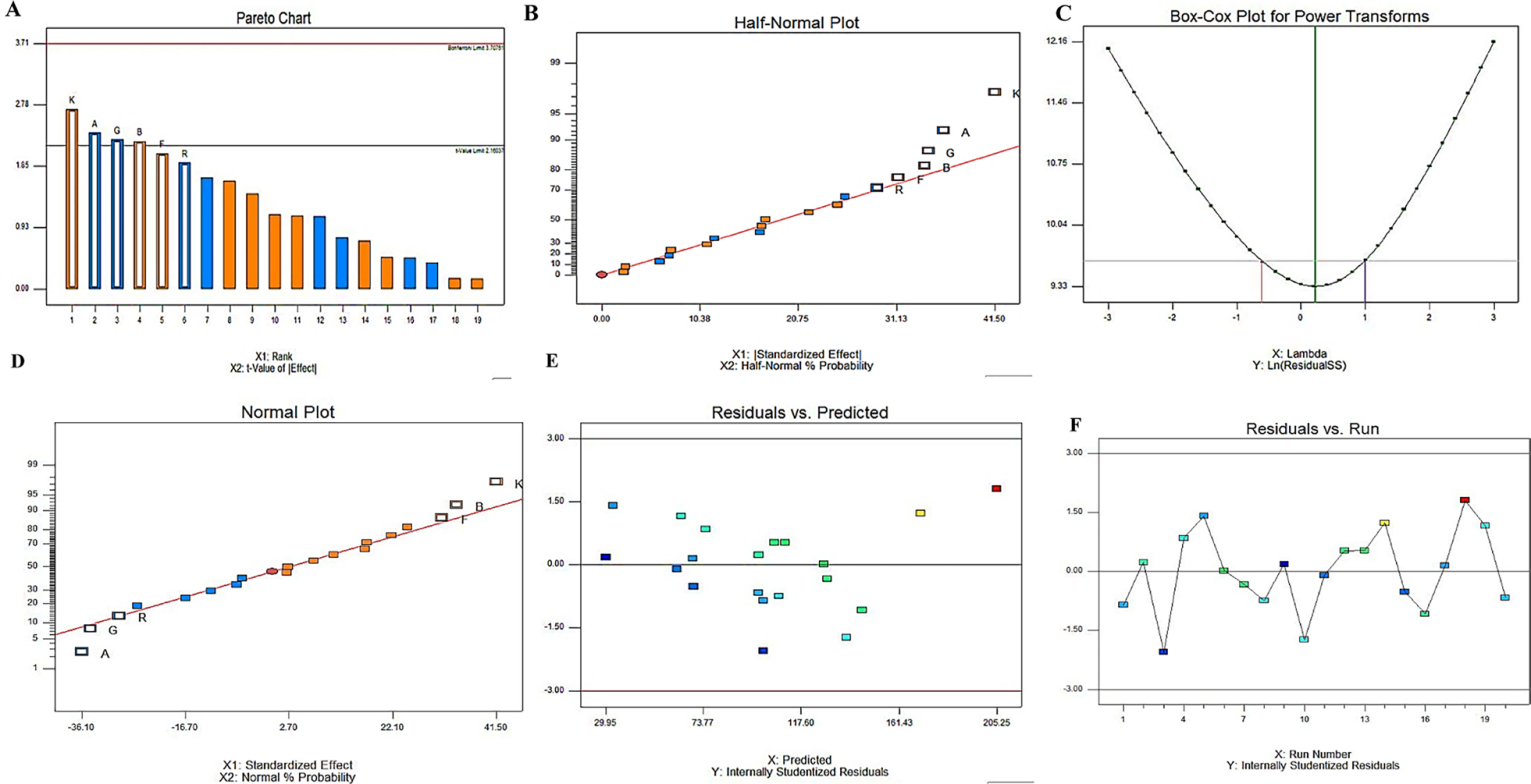
The main effects of different variables on optimization of Taxol production by *A. nigricolor* by Plackett–Burman experimental design. The normal probability plots of the variables for Taxol production by *A. nigricolor* were determined by the first-order polynomial equation. **A** Pareto chart illustrates the order of significance of each variable. **B** Half -Normal plot. **C** Box-Cox power transform. **D** Plot of standardized effect with normal probability. **E** Plot of correlation of the predicted Taxol yield and residuals. **F** Plot of standardized run with normal residuals

The optimization of nutritional requirements for *A. nigricolor* Taxol production was conducted using response surface methodology. The tested 19 variables representing the diverse carbon, nitrogen precursors of Taxol biosynthesis, growth modulators and elicitors were screened by Plackett–Burman design as described in Table 2. The matrix of Plackett–Burman with the actual, and predicted response of Taxol productivity by *A. nigricolor*, was shown in Table 3. The statistical analysis of model exhibited an F-value of 5.13 with 0.66% noise, “P > F” values less than 0.050, ensuring the model significant. The R-square of 0.2975 is in a rational coincident with the "Adj. R-Squared" of 0.5662. The adequate precision metric evaluates the signal-to-noise ratio, with a ratio more than 4 being considered desirable. A value of 8.664 for "Adeq. Precision" demonstrates that the signal is sufficiently strong in addition to the significance of variable was evaluated through the utilization of the *p*-value, and student's t-test, as summarized in Table 4. The results of screening for Taxol production by *A. nigricolor* were visualized through Pareto Chart, normal plot of residuals, and half-normal plot, as depicted in Fig. 5. The probability plots of the experimented variables revealed that five independent factors-phenylalanine, maltose, ammonium tartrate, lactose, and yeast extract, significantly influenced Taxol productivity (Fig. 5). In contrast, the remaining factors exhibited an adverse effect. The residuals' distribution along the diagonal line ensures presence of the independent normality among the tested variables, authenticating the particular alliance of predicted and actual productivity of Taxol. The maximum productivity of Taxol by *A. nigricolor* was recorded in run #18 with actual (205.5 µg/l) and predicted (170.2 µg/l) amounts*.* Using the design of Plackett–Burman, the Taxol yield by *A. nigricolor* was improved by approximately 2 folds related to control (105.2 µg/l). The polynomial equation for Taxol yield by *A. nigricolor* ensures the significance of the independent variables:

**Table 2:** The lowest and highest values of the selected parameters for screening of Taxol production by *A. nigricolor,* with the Plackett Burman Design

No	Variables (g /L)	Value	
		Low (**− **1)	High (+ 1)
X1	Maltose	2.0	6.0
X2	Lactose	2.0	6.0
X3	Sucrose	2.0	6.0
X4	Peptone	3.0	8.0
X5	Soy tone	3.0	8.0
X6	Yeast extract	3.0	6.0
X7	Ammonium tartrate	2.0	4.0
X8	Sodium acetate	2.0	4.0
X9	Cysteine	3.0	6.0
X10	Phenyl alanine	1.0	3.0
X11	Methionine	2.0	4.0
X12	Glycine	2.0	4.0
X13	Sodium nitrate	1.0	3.0
X14	Calcium chloride	1.0	3.0
X15	Magnesium sulphate	0.5	2.0
X16	Potassium hydrogen phosphate	1.0	4.0
X17	Fluconazole	1.0	3.0
X18	Methyl jasmonate	0.1	1.0
X19	Ammonium sulphate	2.0	5.0

**Table 3:** Matrix of Plackett–Burman experimental design for Taxol production by *Amesia nigricolor*

Run	X1	X2	X3	X4	X5	X6	X7	X8	X9	X10	X11	X12	X13	X14	X15	X16	X17	X18	X19	Taxol yield (µg/L)	Predicted Taxol yield (µg/L)	Residuals
1.	1	− 1	− 1	− 1	1	1	1	− 1	1	1	− 1	− 1	1	1	1	1	1	1	− 1	77.8	105.3	− 27.5
2.	1	1	1	1	− 1	1	− 1	1	− 1	− 1	− 1	− 1	1	1	− 1	1	1	− 1	− 1	105.7	99.9	5.8
3.	− 1	− 1	− 1	− 1	1	1	− 1	1	1	− 1	− 1	1	1	1	1	− 1	1	− 1	1	44.8	106.1	− 61.3
4.	1	− 1	1	− 1	1	− 1	− 1	− 1	− 1	1	1	− 1	1	1	− 1	− 1	1	1	1	98.6	74.5	24.1
5.	1	− 1	− 1	− 1	− 1	− 1	− 1	1	1	− 1	1	1	− 1	− 1	1	1	1	1	− 1	72.1	34.2	37.9
6.	1	1	1	1	− 1	1	− 1	− 1	− 1	− 1	1	1	− 1	1	1	− 1	− 1	1	1	128.3	127.5	0.8
7.	− 1	1	1	− 1	− 1	1	1	1	1	− 1	1	− 1	1	− 1	− 1	− 1	− 1	1	1	120.2	129.2	− 9
8.	− 1	− 1	1	1	− 1	1	1	− 1	− 1	1	1	1	1	− 1	1	− 1	1	− 1	− 1	87.5	107.5	− 20
9.	1	− 1	− 1	1	1	1	1	− 1	1	− 1	1	− 1	− 1	− 1	− 1	1	1	− 1	1	35.9	29.5	6.4
10.	1	1	− 1	1	1	− 1	− 1	1	1	1	1	− 1	1	− 1	1	− 1	− 1	− 1	− 1	90.9	137.9	− 47
11.	1	1	1	− 1	1	− 1	1	− 1	− 1	− 1	− 1	1	1	− 1	1	1	− 1	− 1	1	59.2	61.9	− 2.7
12.	1	1	− 1	− 1	1	1	1	1	− 1	1	− 1	1	− 1	− 1	− 1	− 1	1	1	− 1	120.6	105.6	15
13.	− 1	1	− 1	− 1	− 1	− 1	1	1	− 1	1	1	− 1	− 1	1	1	1	1	− 1	1	125.8	110.3	15.5
14.	− 1	− 1	1	1	1	1	− 1	1	− 1	1	− 1	− 1	− 1	− 1	1	1	− 1	1	1	200.8	175.2	30.6
15.	1	− 1	1	1	− 1	− 1	1	1	1	1	− 1	1	− 1	1	− 1	− 1	− 1	− 1	1	55.4	69.3	− 13.9
16.	− 1	1	− 1	1	− 1	− 1	− 1	− 1	1	1	− 1	1	1	− 1	− 1	1	1	1	1	115.2	144.5	− 29.3
17.	− 1	1	1	1	1	− 1	1	− 1	1	− 1	− 1	− 1	− 1	1	1	− 1	1	1	− 1	73.3	86.7	− 13.4
18.	− 1	1	1	− 1	1	1	− 1	− 1	1	1	1	1	− 1	1	− 1	1	− 1	− 1	− 1	205.5	170.2	35.3
19.	− 1	− 1	− 1	1	1	− 1	1	1	− 1	− 1	1	1	1	1	− 1	1	− 1	1	− 1	96.3	102.3	− 6
20.	− 1	− 1	− 1	− 1	− 1	− 1	− 1	− 1	− 1	− 1	− 1	− 1	1	− 1	− 1	− 1	− 1	− 1	− 1	80.6	98.2	− 17.6

**Table 4:** Regression statistics and analysis of variance (ANOVA) for the Placket-Burman design

Source	Sum of squares	Df	Mean Square	F Value	p-Value Prob > F	
Model	36025.1	6	6004.18	5.13	0.0066	
A-Maltose	6516.05	1	6516.05	5.57	0.0346	
B-Lactose	5814.05	1	5814.05	4.97	0.044	
F-Yeast Extract	4898.45	1	4898.45	4.19	0.0615	
G-Am. tartarate	5951.25	1	5951.25	5.09	0.042	Significant
K-Phenylalanine	8611.25	1	8611.25	7.36	0.0177	
R-Fluconazole	4234.05	1	4234.05	3.62	0.0795	
Residual	15205.85	13	1169.68	–	–	
Cor Total	51230.95	19	–	–	–	

	Coefficient		Standard Error	95% CI		VIF
Factor	Estimate	Df		Low	High	
Intercept	101.95	1	7.65	85.43	118.47	
A-Maltose	− 18.05	1	7.65	− 34.57	− 1.53	1
B-Lactose	17.05	1	7.65	0.53	33.57	1
F-Yeast Extract	15.65	1	7.65	− 0.87	32.17	1
G-Am. tartarate	− 17.25	1	7.65	− 33.77	− 0.73	1
K-Phenylalanine	20.75	1	7.65	4.23	37.27	1
R-Fluconazole	− 14.55	1	7.65	− 31.07	1.97	1

Taxol yield by *A. nigricolor* = 96.35- 9.025 ∗ Maltose + 8.525 ∗ Lactose + 10.433 ∗ Yeast extract− 17.25 ∗ ammonium tartarate + 20.75* Phenylalanine.

### Taxol biosynthetic stability with the fungal storage; restoring its biosynthetic machinery

**Fig. 6:**
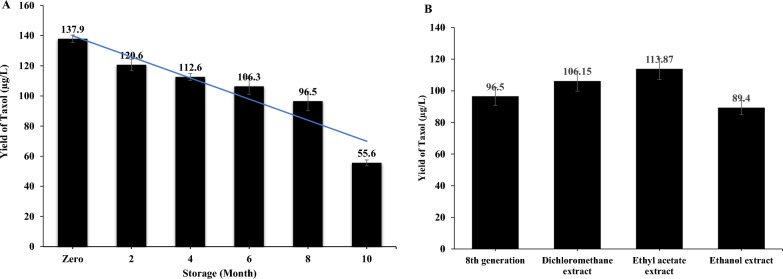
Biosynthetic stability of Taxol by *A. nigricolor* in response to storage and effect of plant extracts on restoring its biosynthetic machinery. **A** The productivity of Taxol for *A. nigricolor* with the fungal preservation as slope culture. **B** The yield of Taxol by *A. nigricolor* in response to amendment with different organic extracts of *E. bubalinus*

Taxol production by *A. nigricolor* upon cultural storage at 4 °C for 10 months was assessed, compared to the zero cultures. From the profile of Taxol productivity, as shown in Fig. 6, a gradual decrease on the Taxol productivity over 10 months was observed. After 6 months of fungal storage at 4 °C, the Taxol productivity of *A. nigricolor* was slightly decreased by 1.3 folds (106.3 μg/l), normalized to zero culture (137.9 μg/l). While the Taxol yield by *A. nigricolor* was slightly inhibited by 2.4 folds by 10th month of storage (55.6 μg/l), compared to the zero culture (Fig. 6). This slight decrease in Taxol yield for *A. nigricolor* with the fungal storage, was coincide with the traits of secondary metabolites synthesis by fungi [13, 37].

This effect of diverse organic extracts, specifically dichloromethane, ethylacetate, and ethanol of *E. bubalinus* leaves on restoring Taxol yield by *A. nigricolor* was assessed, by amending the 5 days cultures of the 8th month storage with the ethanol, dichloromethane, and ethyl acetate extracts of *E. bubalinus* leaves, re-incubated for 14 days. Taxol yield of the 8th months stored *A. nigricolor* (96.5 μg/l), was returned by 1.2 folds with *E. bubalinus* ethylacetate extract (113.8 μg/l) followed by dichloromethane (106.1 μg/l), with undetectable effect by ethanolic extract (Fig. 6).

## Discussion

The potential for commercial Taxol production through endophytic fungi is enhanced by their reliable biosynthetic machinery, rapid growth, and amenability to culture and metabolic manipulation [12, 15, 18]. The practical implementation of fungi commercial production of Taxol has been confronted by the defeat of Taxol productivity of the endophytic fungi with the storage time [9, 17, 20, 53]. Several assumptions were proposed to declare the reduction of Taxol yield by the endophytic fungi, with evidence suggesting that their Taxol biosynthetic machinery depends on signals from the plants or their microbiome to trigger the expression of the biosynthetic genes of Taxol [12, 15, 18]. Several endophytic fungi were reported as potential Taxol producers [11, 12, 14, 15, 54]. However, the common feature is the reduction of Taxol yield with the time of storage. So, the objective of this work was to get an isolate with reliable sustainability for Taxol production. The ethnopharmacolog-ically important plant *E. bubalinus* was selected for this work because its fungal endophytic profile remains unclear, suggesting the potential presence of novel endophytes with desired biosynthetic stability. The cytotoxicity of the ethylacetate extracts of the fungi were preliminary checked from their antimicrobial activity, since most of the cytotoxic compounds have an antimicrobial activity [13, 47, 55]. The ethylacetate extract of *A. nigricolor* derived from *E. bubalinus* demonstrated superior antifungal efficacy against *A. flavus* as the most prevalent opportunistic human pathogen, with significant morbidity and mortality through invasive and superficial infections [13, 56]. *Amesia nigricolor* OR364127.1*,* endophyte of *E. bubalinus,* had the maximum yield for Taxol (105.2 μg/l), which is the first record for this endophytic isolate for Taxol production. The yield of Taxol by *A. nigricolor* was consistent with those reported for *A. niger,* endophyte of *E. whitelockii* [13], *Penicillium polonicum,* endophyte of *Ginkgo biloba* [51], *A. flavipes* (El-Sayed et al., 2019), *Chaetomium* sp [57], *Cladosporium cladosporioides* [58], *Fusarium solani* [59], and *A. niger,* endophytes of *Taxus* sp [60]. PCR mining of taxadiene synthase (tds) gene, as rate-limiting enzyme, served as a sign for evaluating the Taxol biosynthetic machinery [61, 62].

The cytotoxic effects of ethylacetate extract and purified Taxol of *A. nigricolor* were assessed towards the HepG2, MCF7, and Caco-2 carcinoma, normalized to Vero cells as control. Based on the IC_50_ value, the ethyl acetate extract showed strong cytotoxic effects against HepG-2, and MCF7 cells (IC_50_ 19.6–21.15 μg/mL), meeting the criterion for cytotoxic agents, which requires values below 20 μg/mL [63]. These results aligned with those reported for crude extracts from *A. niger* from *E. whitelockii* [13] and *Fusarium oxysporum* from *Dendrobium officinale* [64]. The purified *A. nigricolor* Taxol had a greater activity against HepG2 and MCF7 cells (IC_50_ 0.019–0.023 μM), exhibiting a significantly higher potency compared to Taxol of *P. polonium* (IC_50_, 4.06–6.07 μM) [51]. Coincident results were reported for the Taxol from different fungi [12, 13, 65–68].

The purified *A. nigricolor* Taxol significantly affected HepG2 and MCF-7 cell migration, with wound closure reaching 60% and 64% after 72 h compared to controls, confirming the intervention with the cellular division and matrix formation, inhibiting the metastasis of tumor cells, as matched with the previous studies [13, 60, 69]. The purified *A. nigricolor* induce the cellular apoptosis of HepG-2 by 12 folds, with maximum cellular halt at G2/M phase of cellular growth. This aligns with Taxol's known mechanism of inhibiting functional spindle formation during metaphase in mitotic division, disrupting normal microtubule network reorganization, and inducing apoptosis and cell mortality at G2/M phase [70, 71]. The antiproliferative, anti-wound healing, and apoptotic properties of the purified Taxol of *A. nigricolor* ensure the identical molecular stereochemistry and structural activity relationship of the purified sample compared to the standard one from *T. brevifolia*.

Taxol production by *A. nigricolor* was maximized by Response Surface Methodology, with an overall 2 folds increment over the control that was in coincidence with the previous studies [13, 20, 51, 54, 72]. Actually, the yield of Taxol by *A. nigricolor* (205.5 µg/l) was two folds more than that of *A. candidus* (112 μg/l) [73], *P. polonicum* (90.5 μg/l) [51], *A. flavus* (88.6 μg/l) and *Chaetomium* sp (77.23 μg/l) [57].

The metabolic stability of fungal Taxol during storage remains a major challenge that impedes the further application of the fungal technology for commercial production of Taxol [12, 18, 20]. Therefore, checking the stability of Taxol production by *A. nigricolor* was the profound objective of this study. The results showed an obvious relative stability of Taxol biosynthetic potency in *A. nigricolor* when stored as slope cultures, compared to previous studies [12, 13, 18, 20]. The Taxol yield by *A. terreus* was reduced by 3.5 folds after 6 months’ storage (89 μg/l), normalized to zero culture (270 μg/l) [12, 54], in contrary to the less than 1.2 reduction in Taxol yield by *A. nigricolor*, indicating the relative stability of the biosynthetic machinery, which may be moderately relied on the surrounding microbiome, or the plant signals. However, the productivity of Taxol by *A. nigricolor* was partially restored upon the addition of the ethylacetate extracts of *E. bubalinus,* suggesting the incidence of specific compounds that trigger the expression of the Taxol encoding gene cluster [13, 73]

In conclusion, *Amesia nigricolor* OR364127.1, endophyte of *E. bubalinus*, demonstrated the maximum Taxol productivity. The chemical structure of the purified Taxol was validated by HPLC, FT-IR, UV spectral pattern, and LC-M/MS, compared to the authentic one. The activity of the purified Taxol was verified from the antiproliferative, anti-wound healing, cell cycle and apoptotic activity, ensuring the similar stereochemistry with the authentic one. The stability of Taxol productivity by *A. nigricolor* was assessed, displaying a relative sustainable productivity of Taxol until the 6th month, suggesting the intrinsic stability of Taxol biosynthetic machinery that could be related to the plant-derived metabolites and/or microbiome-associated chemical mediators. So, further transcriptomic and metabolomic analyses are on-going to emphasize the stability of the biosynthetic machinery of Taxol by *A. nigircolor* that could be a new platform for industrial Taxol production.

## Data Availability

All the data are provided in the manuscript.
